# Tumor Metabolic Reprogramming and Ferroptosis: The Impact of Glucose, Protein, and Lipid Metabolism

**DOI:** 10.3390/ijms252413413

**Published:** 2024-12-14

**Authors:** Keyu Zhu, Yuang Cai, Lan Lan, Na Luo

**Affiliations:** 1 School of Medicine, Nankai University, Tianjin 300071, China; 2110402@mail.nankai.edu.cn (K.Z.); 2112041@mail.nankai.edu.cn (Y.C.); 2 School of Pharmaceutical Sciences, South-Central Minzu University, Wuhan 430074, China; 202121131063@mail.scuec.edu.cn

**Keywords:** tumor metabolic reprogramming, ferroptosis, glucose metabolism, protein metabolism, lipid metabolism

## Abstract

Ferroptosis, a novel form of cell death discovered in recent years, is typically accompanied by significant iron accumulation and lipid peroxidation during the process. This article systematically elucidates how tumor metabolic reprogramming affects the ferroptosis process in tumor cells. The paper outlines the basic concepts and physiological significance of tumor metabolic reprogramming and ferroptosis, and delves into the specific regulatory mechanisms of glucose metabolism, protein metabolism, and lipid metabolism on ferroptosis. We also explore how complex metabolic changes in the tumor microenvironment further influence the response of tumor cells to ferroptosis. Glucose metabolism modulates ferroptosis sensitivity by influencing intracellular energetic status and redox balance; protein metabolism, involving amino acid metabolism and protein synthesis, plays a crucial role in the initiation and progression of ferroptosis; and the relationship between lipid metabolism and ferroptosis primarily manifests in the generation and elimination of lipid peroxides. This review aims to provide a new perspective on how tumor cells regulate ferroptosis through metabolic reprogramming, with the ultimate goal of offering a theoretical basis for developing novel therapeutic strategies targeting tumor metabolism and ferroptosis.

## 1. Introduction

Tumor is a disease state induced by multiple carcinogenic factors, in which a cell within a local tissue of the body loses its normal growth control at the genetic level. This loss of control leads to clonal abnormal proliferation of cells, ultimately resulting in the formation of a new biological structure, which we call a tumor. This disease poses a serious challenge to human health, and its profound impact cannot be ignored. Although significant progress has been made in the diagnosis and treatment of tumors in recent decades, unfortunately, tumors remain one of the leading causes of human death [1].

Traditional tumor treatments primarily consist of surgical intervention, chemotherapy, and radiotherapy. However, emerging therapeutic approaches now include endocrine therapy, targeted therapy, and epigenetics-based treatments [2,3]. Surgical intervention, which involves the physical removal of solid tumors, remains the most effective treatment for most solid tumors. Nevertheless, its efficacy is limited when dealing with non-solid tumors, infiltrating tumors, or metastatic lesions [4]. Chemotherapy primarily kills tumor cells by utilizing chemical substances to block DNA synthesis, damage DNA molecules, and interfere with cell division [5]. Radiotherapy, on the other hand, employs high-energy rays such as X-rays, gamma rays, protons, and neutrons to damage intracellular molecules like DNA and proteins, thereby killing tumor cells and achieving the goal of tumor treatment [6]. It is important to note that both chemotherapy and radiotherapy lack specificity, meaning that they inhibit the growth of not only tumor cells but also normal cells. This non-specificity can lead to adverse reactions such as myelosuppression and liver function damage [7]. Endocrine therapy, targeted therapy, and epigenetics-based treatments are carefully designed, taking into account the dependency of certain tumors on specific hormones, the pivotal proteins involved in the onset and progression of the disease, and the epigenetic regulation of tumor genes. These modern approaches exhibit a marked degree of specificity compared to traditional treatment methods, thereby causing fewer adverse reactions due to reduced damage to healthy cells [8]. Simultaneously, a growing number of studies have indicated that tumor cells can develop resistance to an increasing array of chemotherapy drugs and radiation therapy, elevating the likelihood of treatment failure and recurrence [9,10]. Therefore, discovering novel pathways that regulate cell death and exploring their feasibility in tumor treatment bears significant clinical and practical importance.

Tumor metabolic reprogramming refers to the metabolic adaptations made by tumor cells in response to various stresses. The metabolism of tumor cells differs significantly from that of normal cells of the same origin. The biochemical abnormalities in tumor cell metabolism were first discovered by the biochemist Otto Warburg, who observed that tumor cells maintain vigorous glycolysis and consume large amounts of glucose even under conditions of adequate oxygen supply. This abnormal metabolic pattern is known as the “Warburg effect” [11]. Tumor metabolic reprogramming encompasses reprogramming of glucose metabolism, amino acid metabolism, and lipid metabolism [12]. The mechanisms underlying tumor metabolic reprogramming are complex, and the specific reasons for this reprogramming have not been fully elucidated as of yet. Current research has revealed that through metabolic reprogramming, tumors not only obtain adequate energy supply but also balance the synthesis of biological macromolecules, ultimately achieving rapid tumor expansion [13].

Ferroptosis, a form of programmed cell death first named by Stockwell, involves an iron-dependent process [14]. Specifically, intracellular free ferrous iron (Fe^2+^) participates in the Fenton reaction, generating reactive oxygen species (ROS) represented by hydroxyl radicals. The accumulation of ROS oxidizes polyunsaturated fatty acids, forming lipid peroxides represented by malondialdehyde (MDA). MDA causes damage and rupture to the cell membrane, ultimately leading to ferroptosis [15]. The key positive regulatory mechanism of ferroptosis lies in the formation of lipid peroxides, while the negative regulatory mechanism involves the elimination of these peroxides. The hallmark morphological features of ferroptosis include the reduction or disappearance of mitochondrial cristae, shrinkage and rupture of the mitochondrial outer membrane, and deepening of mitochondrial staining. Ferroptosis differs from apoptosis, necrosis, and autophagy in terms of molecular regulation and characteristic cellular morphological changes [16].

**Figure 1:**
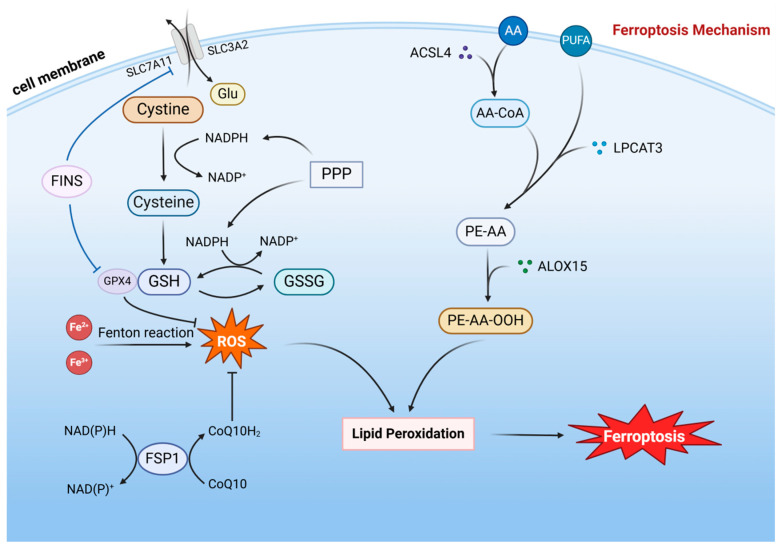
Ferroptosis mechanism: Ferroptosis is directly triggered by lipid peroxides, with iron ions catalyzing the Fenton reaction to generate reactive oxygen species (ROS) that lead to the formation of lipid peroxides. Polyunsaturated fatty acids (PUFAs) and arachidonic acid (AA) in the cell membrane can undergo a series of catalytic reactions to form peroxidized lipids, ultimately resulting in ferroptosis. Glutathione peroxidase 4 (GPX4) plays a pivotal role in inhibiting ferroptosis by consuming glutathione (GSH) to scavenge ROS. The Xc-system facilitates GSH synthesis by transporting glutamate and cystine, thereby suppressing ferroptosis. Ferroptosis inducers (FINs) promote ferroptosis by inhibiting both the Xc-system and GPX4. Ubiquinone (CoQ10), under the catalysis of ferroptosis suppressor protein 1 (FSP1), can be converted into its reduced form, CoQ10H2, which scavenges ROS and thus inhibits ferroptosis. (Abbreviations: SLC7A11, solute carrier family 7 member 11; SLC3A2, solute carrier family 3 member 2; PPP, pentose phosphate pathway; ACSL4, acyl-CoA synthetase long-chain family member 4; LPCAT3, lysophosphatidylcholine acyltransferase 3; ALOX15, arachidonate 15-lipoxygenase.) Created in BioRender.com (accessed on 9 September 2024).

In recent years, an increasing number of researchers have focused on the role of ferroptosis in cancer, aiming to promote ferroptosis by enhancing the formation of lipid peroxides and inhibiting their elimination, thereby suppressing the proliferation of tumor cells [17]. Researchers have now developed various ferroptosis-inducing agents (FINs) that can promote ferroptosis in tumors by inhibiting key proteins that negatively regulate this process, such as glutathione peroxidase 4 (GPX4) and solute carrier family 7 member 11 (SLC7A11, also known as xCT). FINs have been found to exhibit inhibitory effects on multiple types of tumors [18]. Simultaneously, FINs can be combined with conventional tumor therapies to enhance treatment efficacy (Figure 1). Studies have demonstrated that the combination of FINs targeting GPX4 and SLC7A11 inhibition with radiotherapy can increase tumor cell ferroptosis, thereby augmenting the tumor-killing effects of radiation [19]. FINs targeting SLC7A11 can enhance the sensitivity of cancer cells to chemotherapy drugs [20]. Ferroptosis is closely linked to cellular glucose metabolism, amino acid metabolism, and lipid metabolism. Due to metabolic reprogramming in tumors, their ferroptosis profile significantly differs from that of normal cells.

This article will provide a comprehensive review of the role of tumor metabolic reprogramming in ferroptosis, offering new insights into the study of tumor metabolism and ferroptosis.

## 2. The Impact of Glucose Metabolism on Ferroptosis

Glucose serves as the primary energy source and fundamental material for biomacromolecule synthesis in most cells. Under normal circumstances, glucose is converted into pyruvate through glycolysis, followed by complete oxidative decomposition via the citric acid cycle and oxidative phosphorylation to generate energy, water, and carbon dioxide. Under hypoxic conditions, pyruvate is converted into lactic acid through the catalytic action of lactate dehydrogenase. Concurrently, glucose-6-phosphate can enter the pentose phosphate pathway under the catalysis of glucose-6-phosphate dehydrogenase, generating NADPH and other biomolecules [21]. NADPH serves as a crucial biological reducing agent, playing a vital role in maintaining the redox homeostasis of cells [22,23].

### 2.1. Glucose Metabolism and Ferroptosis in Normal Cells

#### 2.1.1. Oxidative Phosphorylation Generates Reactive Oxygen Species (ROS), Promoting Ferroptosis

**Figure 2:**
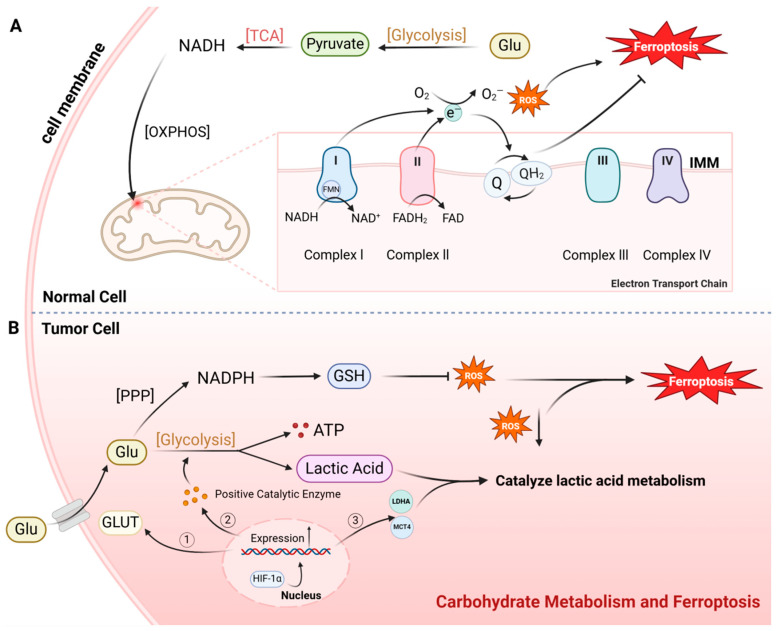
Carbohydrate metabolism and ferroptosis: (**A**) In normal cells, glucose (Glu) primarily undergoes complete oxidative decomposition. Within the electron transport chain of the mitochondrial inner membrane, electrons from complex I and complex II can occasionally transfer to oxygen molecules, generating reactive oxygen species (ROS) that induce ferroptosis. Concurrently, coenzyme Q (CoQ) in the electron transport chain scavenges some ROS, thereby inhibiting ferroptosis. (**B**) In tumor cells, Glu metabolism is primarily glycolytic. Hypoxia-inducible factor 1 subunit alpha (HIF-1α) promotes the expression of glucose transporters (GLUT), glycolytic enzymes, and lactate metabolism enzymes, thereby enhancing glycolysis in tumor cells. Consequently, the formation of ROS through complete glucose oxidation in the electron transport chain is reduced, leading to a decrease in ferroptosis. Additionally, tumor cells shunt more glucose into the pentose phosphate pathway (PPP), generating NADPH to further inhibit ferroptosis. (Abbreviations: OXPHOS, oxidative phosphorylation; FAD, flavin adenine dinucleotide; IMM, inner mitochondrial membrane; LDHA, lactate dehydrogenase A; MCT4, monocarboxylate transporter 4.) Created in BioRender.com (accessed on 9 September 2024).

The complete oxidative decomposition of sugar promotes ferroptosis by increasing the production of reactive oxygen species (ROS). This process involves glycolysis, the tricarboxylic acid cycle, and ultimately the formation of NADH, which enters the electron transport chain in the mitochondrial inner membrane. The electron transport chain consists of complexes I, II, III, and IV, with complex I being the most prone to generating ROS during electron transfer. Complex I receives electrons from NADH and passes them to flavin mononucleotide (FMN). These electrons are then transferred through a series of iron–sulfur proteins to coenzyme Q (CoQ) and beyond. Oxygen can capture electrons from NADH at the FMN site of complex I, leading to the formation of ROS [24]. Studies indicate that the R site within complex I serves as a critical location where oxygen binds to electrons, leading to the formation of ROS [24]. In conclusion, mitochondria produce ROS during the oxidative phosphorylation process of glucose metabolism. An excess of ROS can trigger lipid peroxidation of both the mitochondrial and cytoplasmic membranes, ultimately inducing ferroptosis (Figure 2A).

#### 2.1.2. Oxidative Phosphorylation Generates Coenzyme Q (CoQ), Which Inhibits Ferroptosis

Coenzyme Q (CoQ) exists in three redox forms on the mitochondrial membrane: oxidized, reduced, and the intermediate semiquinone radical. As a lipid-soluble compound, CoQ can freely move and carry electrons across the mitochondrial membrane. Within the electron transport chain, CoQ accepts electrons from complexes I and II, ultimately transferring them to complexes III and IV. Electron-enriched CoQ not only serves as a crucial molecule in electron transport but also functions as a significant reducing agent capable of scavenging peroxides and inhibiting ferroptosis [25]. Kirill Bersuker and colleagues have discovered that reduced CoQ acts as a novel molecule that inhibits ferroptosis independently of the GPX4 pathway [26]. They first identified that FSP1 (ferroptosis suppressor protein 1) can promote the conversion of oxidized CoQ to its reduced form, thereby scavenging lipid peroxides and ultimately suppressing the occurrence of ferroptosis. This CoQ pathway is closely associated with ferroptosis in various cancer cells, including lung cancer. An increase in FSP1 expression enhances the resistance of tumors to ferroptosis [26] (Figure 2A).

#### 2.1.3. Pentose Phosphate Pathway Inhibits Ferroptosis

The pentose phosphate pathway (PPP) is a crucial branch of glycolytic metabolism, where glucose-6-phosphate undergoes a series of transformations to produce pentose phosphates and NADPH. Pentose phosphates serve as precursors for nucleotides and coenzymes, while NADPH functions as a vital bioreductant within the cell, maintaining intracellular redox homeostasis. NADPH can inhibit ferroptosis in multiple ways. Specifically, NADPH acts as an electron carrier, transferring electrons to glutathione disulfide (GSSG) under the catalytic action of glutathione reductase (GR), thereby reducing it to glutathione (GSH) [27].

Under the catalysis of glutathione peroxidase 4 (GPX4), GSH prevents lipids from being peroxidized by reactive oxygen species (ROS) and eliminates lipid peroxides, thus reducing the occurrence of ferroptosis [28]. Additionally, NADPH aids in maintaining the reduced state of CoQ, preserving its ability to scavenge lipid peroxides and inhibiting the onset of ferroptosis. Furthermore, NADPH is essential for the breakdown of cystine into cysteine, which is one of the fundamental precursors for GSH synthesis. Studies have also indicated that NADPH sustains the cellular reduced state through thioredoxin (Trx) [28]. NADPH maintains the reducing power of thioredoxin (Trx), and the reduced form of Trx can scavenge intracellular peroxides. According to Evijola Llabani and colleagues, the use of small molecule compounds to target and inhibit Trx can promote the occurrence of ferroptosis in cells [29].

### 2.2. Glucose Metabolism and Ferroptosis in Tumor Cells

#### 2.2.1. Increased Metabolism of the Glycolysis in Tumor Cells

Tumor cells reduce oxidative phosphorylation by increasing glycolysis, limiting the production of reactive oxygen species (ROS) and thereby inhibiting ferroptosis. Although tumor cells obtain less energy from aerobic glycolysis compared to complete oxidative decomposition of glucose, this metabolic pathway effectively avoids the combination of oxygen molecules with electrons, restricting the excessive generation of ROS. Current research indicates that due to their faster metabolism compared to normal cells, tumor cells exhibit higher ROS levels. Moderate amounts of ROS can induce DNA mutations, leading to the emergence of oncogenic gene variants. However, excessive ROS levels can cause cytotoxicity, ultimately resulting in cell death [30].

The activation of hypoxia-inducible factor 1α (HIF1α) is a critical factor in regulating glycolysis in tumor cells. As a transcription factor induced by hypoxic environments, HIF1α can influence the transcription of multiple key proteins in the glycolytic pathway. Glucose transporter (GLUT) is a channel protein located on the cell membrane responsible for transporting glucose into the cell. Studies have shown that HIF1α can promote the expression of GLUT1 and GLUT3, thereby increasing glucose transport into cells. In gliomas, bladder cancer, and various other tumors, the expression levels of HIF1α, GLUT1, and GLUT3 are elevated, and these levels are correlated with tumor prognosis [31,32]. HIF1α enhances the glycolytic rate by increasing the expression of enzymes involved in the glycolytic pathway, enabling more glucose to be degraded through glycolysis [33]. Studies have shown that all 12 enzymes in the glycolytic pathway are positively regulated by HIF1α [33]. Additionally, HIF1α upregulates the expression of lactate dehydrogenase (LDHA) and monocarboxylate transporter 4 (MCT4). LDHA catalyzes the conversion of pyruvate to lactate, while MCT4 facilitates the efflux of lactate from the cell. Thus, HIF1α promotes the formation and efflux of lactate from pyruvate, ensuring the continuous progression of glycolysis [34] (Figure 2B).

The PI3K/AKT pathway also regulates glycolysis in tumor cells. Mutations in the tumor suppressor gene *pten* in tumor cells lead to abnormal activation of the PI3K/AKT pathway. As a crucial protein regulating aerobic glycolysis in tumor cells, the activation of AKT1 can increase GLUT expression and maintain the activity of hexokinase and phosphofructokinase 2 during glycolysis [35], resulting in tumor cells utilizing more glucose for aerobic glycolysis.

In summary, tumor cells promote glycolysis and reduce oxidative phosphorylation through a series of regulatory mechanisms, limiting the excessive production of ROS. This, in turn, reduces the generation of intracellular lipid peroxides and the occurrence of ferroptosis.

#### 2.2.2. Increased Metabolism of the Pentose Phosphate Pathway in Tumor Cells

Due to their rapid proliferation, tumor cells exhibit significantly faster metabolism and produce higher levels of reactive oxygen species (ROS) compared to normal cells [36]. To prevent excessive ROS from inducing tumor cell death, these cells demonstrate enhanced antioxidant capabilities, with an increase in the pentose phosphate pathway (PPP) significantly boosting their antioxidant capacity [37]. Research indicates that various tumor cells, including those in breast cancer and lung cancer, show an upregulation of the PPP. This upregulation provides tumor cells with a greater supply of pentose phosphates and NADPH. The former serves as a raw material for nucleic acid synthesis during tumor cell proliferation, while the latter plays a crucial role in maintaining cellular redox homeostasis. The elevation of NADPH levels can suppress the occurrence of ferroptosis [36].

Tumor cells can upregulate the rate-limiting enzymes of the pentose phosphate pathway (PPP) to promote its occurrence. Glucose-6-phosphate dehydrogenase (G6PDH) is the first rate-limiting enzyme that catalyzes the PPP, playing a pivotal role in initiating PPP metabolism. The research conducted by Hilf et al. indicates that G6PDH is more prevalent in precancerous tissues and tumors, accounting for 35% of total enzyme activity, whereas it is present in only a small amount (less than 15%) in the mammary glands of pregnant mice and almost absent (approximately 5%) in the mammary glands of lactating mice. This suggests that G6PD is overexpressed in cancers such as mouse breast cancer, thereby promoting the progression of the pentose phosphate pathway (PPP) [38]. Additionally, the consumption of NADPH upregulates the activity of G6PDH. Since tumor cells eliminate a higher amount of reactive oxygen species (ROS) and consume more NADPH, the activity of G6PDH in tumor cells is also higher than that in normal cells [39].

Pyruvate kinase M2 (PKM2) in tumor cells also plays a role in regulating the pentose phosphate pathway (PPP). PKM2 is a crucial enzyme in glycolytic metabolism. Due to the elevated levels of reactive oxygen species (ROS) in tumor cells, the cysteine residues of PKM2 can be oxidized by ROS, leading to a decrease in its catalytic activity. This inhibition of the glycolytic pathway in tumor cells results in a greater flux of glucose into the PPP [40].

In summary, tumor cells enhance the production of NADPH by promoting the pentose phosphate pathway, thereby maintaining cellular redox homeostasis and reducing the formation of intracellular lipid peroxides, ultimately mitigating the occurrence of ferroptosis.

## 3. The Influence of Protein Metabolism on Ferroptosis

### 3.1. Glutathione Synthesis and Its Implications in Ferroptosis

**Figure 3:**
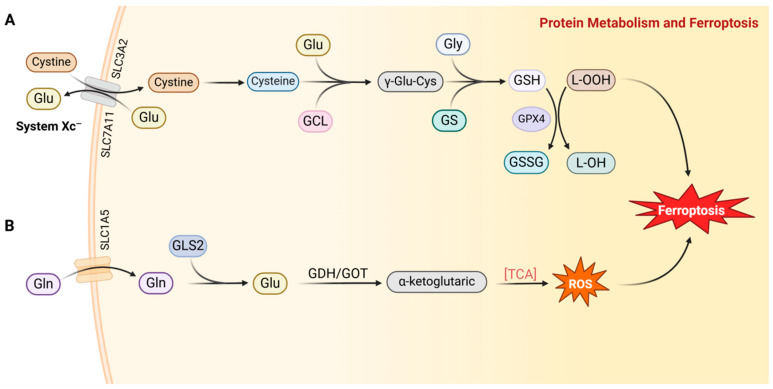
Amino acid metabolism and ferroptosis: (**A**) The Xc-system, comprising SLC7A11 and SLC3A2, transports cystine and glutamate (Glu). Cystine undergoes a series of enzymatic reactions to form glutathione (GSH), which, under the catalysis of glutathione peroxidase 4 (GPX4), scavenges lipid peroxides to inhibit ferroptosis. Inhibition of the Xc-system impairs GSH formation, thereby promoting ferroptosis. (**B**) Glutamine (Gln) is catalyzed by Glutaminase 2 (GLS2) to produce glutamate, which is further catalyzed to form α-ketoglutarate, entering the tricarboxylic acid cycle (TCA) and generating ROS that promote ferroptosis. (Abbreviations: GCL, glutamate cysteine ligase; GS, glutathione synthetase; SLC1A5, solute carrier family 1 member 5; GDH, glutamate dehydrogenase; GOT, glutamic oxaloacetic transaminase.) Created in BioRender.com (accessed on 9 September 2024).

Glutathione (GSH) not only serves as a crucial component for protein synthesis but also plays a pivotal role as an intracellular antioxidant, vital in maintaining cellular redox homeostasis. As a tripeptide, GSH is synthesized through a two-step enzymatic reaction. Initially, cysteine and glutamic acid are linked under the catalysis of glutamate–cysteine ligase (GCL). Subsequently, glycine is incorporated into this structure, catalyzed by glutathione synthetase (GS), to form GSH [41]. Importantly, GPX4 harnesses the antioxidant properties of GSH to scavenge intracellular lipid peroxides, thereby inhibiting the onset of ferroptosis [42]. GPX4 converts GSH to its oxidized form, glutathione disulfide (GSSG), while simultaneously reducing cytotoxic lipid peroxides (L-OOH) to their corresponding non-toxic alcohols (L-OH). This process mitigates the generation of lipid peroxides, inhibits Fe^2+^-dependent ROS formation, and consequently reduces the occurrence of ferroptosis in tumor cells [43,44] (Figure 3A). Studies have revealed that GPX4 is highly expressed in metastatic cancers, closely correlating with tumor progression [45].

### 3.2. The Xc-System and Its Role in Ferroptosis

The rate of glutathione (GSH) synthesis is dependent on the availability of its precursors: cysteine, glutamic acid, and glycine. Endogenous cysteine is primarily generated through the reduction of cystine [46]. The Xc-system consists of two protein chains: the SLC7A11 light chain and the SLC3A2 heavy chain. SLC7A11 serves as the substrate-specific protein in the Xc-system, playing a pivotal role. Functioning as a cystine/glutamate antiporter, the Xc-system transports one molecule of cystine into the cell and exports one molecule of glutamate, thus increasing intracellular cystine content [47] (Figure 3A). The research by Chun-Shik Shin et al. has demonstrated that LC7A11 knockdown cells can significantly maintain their intracellular glutamate levels compared to controls after glucose depletion. Conversely, HeLa cells overexpressing SLC7A11 exhibit lower glutamate levels than control cells [48]. From this, it is inferred that tumor cells overexpressing LC7A11, even when glucose is reduced, can exhibit higher cysteine levels in an Xc-system-dependent manner, promoting GSH synthesis and subsequently reducing the occurrence of ferroptosis, thereby enhancing cell survival.

The research by Lim et al. also confirms that tumor cells overexpress the Xc-system to uptake more cystine as a precursor for GSH production [49]. Zhang et al. discovered that liver cancer cells overexpress SLC7A11, and its expression level correlates with the prognosis of liver cancer [50]. Zhong et al. found that prostate cancer cells also overexpress SLC7A11, and this overexpression is associated with increased radioresistance, invasiveness, and metastatic potential of the cancer cells [51].

Therefore, the Xc-system may become a potential intervention target for tumor therapy. Inhibition of the Xc-system can induce ferroptosis, thereby suppressing tumor development. Yang et al. discovered that metformin promotes ferroptosis in breast cancer cells by inhibiting the ubiquitination of SLC7A11, which destabilizes the Xc-system [52]. Studies have also shown that P53 induces ferroptosis in tumor cells by suppressing SLC7A11 expression, thus inhibiting cystine uptake via the Xc-system [53]. This provides new insights for the development of new tumor treatment methods.

### 3.3. The Glutamine Transport System and Ferroptosis

Glutamine, an essential amino acid in humans, is widely involved in various metabolic activities, including protein synthesis, glutathione synthesis, and energy metabolism. Tumor cells primarily acquire glutamine through two transport proteins, SLC1A5 and SLC38A1. Under the catalytic action of glutaminase (GLS), glutamine is converted into glutamic acid and free ammonia [54]. There are two isoforms of GLS, namely GLS1 and GLS2. The glutamic acid produced by GLS2-catalyzed glutamine can serve as a carbon source for oxidative phosphorylation, thereby promoting the occurrence of ferroptosis [55]. Specifically, glutamic acid catalyzed by GLS2 generates α-ketoglutarate (α-KG) under the catalysis of glutamate dehydrogenase (GDH) or glutamic–oxaloacetic transaminase (GOT). This metabolite enters the tricarboxylic acid cycle for oxidative phosphorylation, resulting in the production of ROS and the induction of ferroptosis [55,56] (Figure 3B). Studies have shown that tumor cells with high metabolic activity in the tricarboxylic acid cycle uptake more glutamine and produce more ROS, making them more susceptible to ferroptosis [57]. Yang et al. found that miR-137 can directly target Gln importer SLC1A5, indirectly inhibit glutamine transport and metabolism by inhibiting SLC1A5 or glutamate oxaloacetate transaminase, and ultimately lead to a reduction in ferroptosis in melanoma cells [58].

Simultaneously, glutamic acid, produced by the catalytic decomposition of glutamine, can also inhibit ferroptosis in tumors. In this metabolic pathway, the enzyme primarily responsible for catalysis is GLS1, rather than GLS2. Research indicates that compared to normal cells, malignant tumors consume more glutamine, and they cannot survive without exogenous glutamine [59]. Additionally, GLS1 is highly expressed in tumor cells such as rectal and prostate cancers, compared to adjacent tissues [60,61]. Glutamic acid, produced by GLS2-catalyzed glutamine, serves as a precursor for glutathione synthesis, playing a crucial role in maintaining cellular redox homeostasis. Simultaneously, as tumor cells excrete glutamic acid during cysteine uptake via the Xc-system, they increase glutamine intake and catabolism to replenish glutamic acid losses, thereby sustaining intracellular GSH synthesis and redox balance [62].

Furthermore, glutamine serves as a precursor for nucleotide biosynthesis. In the absence of glutamine, tumor cells can utilize glutamic acid to generate glutamine under the catalytic action of glutamine synthetase, thereby maintaining nucleotide production and supporting cellular replication [63]. Studies have demonstrated that inhibiting the expression of glutamine synthetase impairs DNA repair in tumor cells, ultimately enhancing their sensitivity to radiotherapy [64].

### 3.4. Protein Catabolism and Ferroptosis

Intracellular proteins are primarily degraded through two systems: the ubiquitin-proteasome system (UPS) and autophagy.

#### 3.4.1. The Ubiquitin–Proteasome System and Ferroptosis

The ubiquitin–proteasome system (UPS) is the primary pathway for intracellular protein degradation, accounting for over 80% of protein breakdown within cells [65]. The UPS consists of ubiquitin (Ub), ubiquitin-activating enzyme (E1), ubiquitin-conjugating enzyme (E2), ubiquitin ligase (E3), the proteasome, and the protein substrate to be degraded. Initially, the protein substrate undergoes ubiquitination through an enzyme cascade involving E1, E2, and E3, which covalently attaches ubiquitin to the protein [66]. Ubiquitination can generate various types of ubiquitin chains, each representing a distinct ubiquitination tag with different functions. The proteasome is an ATP-dependent protein degradation complex where ubiquitin-tagged protein substrates are degraded. Additionally, deubiquitinating enzymes (DUBs) exist within cells to remove ubiquitin from target proteins, thereby preventing their ubiquitination and degradation [65]. The interplay between ubiquitination and deubiquitination regulates intracellular protein catabolism and modulates ferroptosis.

Studies have shown that the deubiquitinating enzyme OTUB1 inhibits ferroptosis by stabilizing SLC7A11 [67]. SLC7A11 is a crucial component of the Xc-system, essential for glutathione synthesis and antioxidant stress, thereby inhibiting ferroptosis. OTUB1, which is overexpressed in multiple human cancers, directly interacts with SLC7A11 to remove its ubiquitination marks, preventing proteasome-mediated degradation and increasing its stability, thus inhibiting the activation of ferroptosis. Research indicates that inhibition of OTUB1 enhances cell sensitivity to erastin-induced ferroptosis [67]. Furthermore, overexpression of the cancer stem cell marker CD44 promotes the interaction between OTUB1 and SLC7A11, reducing SLC7A11 degradation and inhibiting ferroptosis. In summary, tumor cells may inhibit ferroptosis by increasing the expression of the deubiquitinating enzyme OTUB1, which prevents SLC7A11 degradation.

Other studies have demonstrated that inhibiting deubiquitinating enzymes can promote the degradation of GPX4, thereby inducing ferroptosis [68]. Pyridinethione palladium complex (PdPT) is a broad-spectrum inhibitor of deubiquitinating enzymes. GPX4 is a key inhibitory protein in ferroptosis. Research has shown that in small cell lung cancer cells, PdPT inhibits the deubiquitination of GPX4, promoting its proteasome-mediated degradation and inducing ferroptosis [68].

Lactotransferrin (LTF) is an iron-binding transport protein that induces ferroptosis by increasing cellular iron uptake and causing iron dysregulation. Studies have indicated that ubiquitination and degradation of LTF mediated by the ubiquitin ligase NEDD4L can reduce erastin-induced iron accumulation and ferroptosis in human pancreatic and ovarian cancer cells [69].

In hepatocellular carcinoma, research has found that SOCS2 enhances the ubiquitination and degradation of SLC7A11, thereby promoting ferroptosis in tumor cells and increasing radiosensitivity [70]. Specifically, the SH2 domain of SOCS2 specifically recognizes the N-terminal structure of SLC7A11, and SOCS2 can form an SOCS2/elongin B/C complex to recruit ubiquitin molecules, promoting the ubiquitination and degradation of SLC7A11 and ultimately facilitating ferroptosis [70].

#### 3.4.2. Autophagy and Ferroptosis

Autophagy is a process in which cells utilize lysosomes to degrade intracellular molecules such as proteins, carbohydrates, lipids, and nucleic acids [71]. There are three types of autophagy: microautophagy, macroautophagy, and chaperone-mediated autophagy (CMA). Microautophagy involves the direct invagination of the lysosomal membrane to engulf and degrade substrates [72]. Macroautophagy, the most studied type of autophagy, entails the encapsulation of substrate proteins by a double-membrane structure to form autophagosomes. These autophagosomes fuse with the lysosomal membrane to release substrate proteins into the lysosome, where they are degraded by lysosomal enzymes [73]. Chaperone-mediated autophagy does not involve vesicles but relies on the chaperone protein Hsc70 to expose the unique recognition motif KFERQ of target proteins. Since lysosomes contain the receptor protein LAMP2A that can recognize KFERQ, target proteins are specifically transported into lysosomes for degradation. Under environmental stress, appropriate upregulation of autophagy promotes cell survival, but excessive autophagy leads to cell death [74]. Extensive research has demonstrated a close relationship between autophagic protein degradation and ferroptosis.

Ferritinophagy, the autophagic degradation of ferritin, disrupts intracellular iron homeostasis and induces ferroptosis. Ferritin, the iron storage protein in cells, releases iron ions upon degradation, triggering ferroptosis. Nuclear receptor coactivator 4 (NCOA4) is an autophagy receptor that binds to ferritin and promotes its degradation through ferritinophagy [75]. Studies have shown that overexpression of NCOA4 promotes ferritinophagy, leading to intracellular iron dysregulation and ferroptosis, while knockdown of NCOA4 has the opposite effect [76]. Additionally, research indicates that dihydroartemisinin can induce ferritinophagy in leukemia cells, promoting ferroptosis and aiding in cancer treatment [77].

Clockophagy is a novel selective autophagy process where the core circadian rhythm protein ARNTL is selectively degraded through autophagy. ARNTL promotes HIF1A activation by inhibiting Egln2 transcription, thereby inhibiting ferroptosis. Studies have shown that SQSTM1, as an autophagy receptor, mediates the entry of ARNTL into lysosomes for degradation, inhibiting HIF1A activation and inducing ferroptosis [78].

Chaperone-mediated autophagy (CMA) also affects the occurrence of ferroptosis. Research has demonstrated that GPX4, a key inhibitory protein in ferroptosis, contains the KFERQ motif and can be degraded by HSP90-mediated CMA, thereby promoting ferroptosis. This study also showed that the use of the HSP90 inhibitor CDDO can inhibit CMA, reducing GPX4 degradation and subsequently decreasing ferroptosis [79].

Cathepsins in lysosomes are also closely associated with ferroptosis. Studies have shown that increased expression of cathepsin B (CTSB) promotes ferroptosis. Iron-dependent increases in lysosomal membrane permeability cause CTSB to translocate to the nucleus, causing DNA and histone damage and leading to ferroptosis [80]. However, research suggests that lysosome-dependent extracellular protein degradation inhibits ferroptosis in cancer cells [81]. Cancer cells can reduce ferroptosis induced by cystine deprivation by uptake and degradation of extracellular albumin. The mechanism involves the degradation of albumin by CTSB to form cystine, which promotes glutathione synthesis and inhibits oxidative stress and ferroptosis [81].

In summary, the mechanisms of protein degradation are complex and can either promote or inhibit ferroptosis, depending on the protein being degraded. Simply put, if the substrate degraded is a protein that inhibits ferroptosis, it will promote ferroptosis. Conversely, if the substrate degraded is a protein that promotes ferroptosis, it will inhibit ferroptosis.

## 4. Lipid Metabolism and Ferroptosis

Tumor development requires lipids as essential building blocks, and tumor cells often exhibit enhanced absorption or synthesis of fatty acids. A subset of specific tumor types, such as prostate cancer, rely on the energy generated from fatty acid beta-oxidation as their primary energy source [82]. Studies have revealed that key genes associated with fatty acid metabolism pathways are upregulated in tumor cells, and the specific inhibition of these enzymes using inhibitors can suppress tumor growth [83].

Current research indicates that ferroptosis is directly induced by lipid peroxidation. The lipids primarily responsible for triggering ferroptosis are phospholipids (PL), which constitute a major component of cell membranes. Phospholipids consist of a head group and two fatty acid acyl chains (sn1 and sn2), which can be saturated, monounsaturated (MUFAs), or polyunsaturated fatty acids (PUFAs). Among these different types of fatty acids, the bis-allylic positions in PUFAs are unstable and prone to oxidative cleavage, forming free radicals. Consequently, PUFA-PLs containing bis-allylic positions are most susceptible to oxidative cleavage by oxidants, forming phospholipid peroxides. Without the presence of antioxidants, peroxidized lipids can propagate and spread to surrounding lipids [84].

### 4.1. Polyunsaturated Fatty Acids and Ferroptosis

Fatty acids, crucial lipid components within cells, serve not only as significant energy sources but also as the fundamental building blocks of various lipid structures, consisting of a hydrocarbon backbone and a terminal carboxylic acid group. Polyunsaturated fatty acids (PUFA) refer to straight-chain fatty acids containing two or more double bonds and a carbon chain length of 18 to 22 carbon atoms. Arachidonic acid (AA) is a type of omega-6 polyunsaturated fatty acid. Intracellular polyunsaturated fatty acids are key lipids that are oxidized to form lipid peroxides, which induce ferroptosis. Acyl-CoA synthetase (ACS) catalyzes the conversion of free fatty acids into fatty acyl-CoAs, enabling their utilization [85]. Long-chain acyl-CoA synthetase (ACSL), a member of the ACS family, specifically catalyzes the formation of long-chain acyl-CoAs. Among them, ACSL4 is involved in the regulation of arachidonic acid and eicosapentaenoic acid. Specifically, ACSL4 catalyzes the insertion of coenzyme A (CoA) into arachidonic acid, forming arachidonoyl-CoA (AA-CoA) [86].

**Figure 4:**
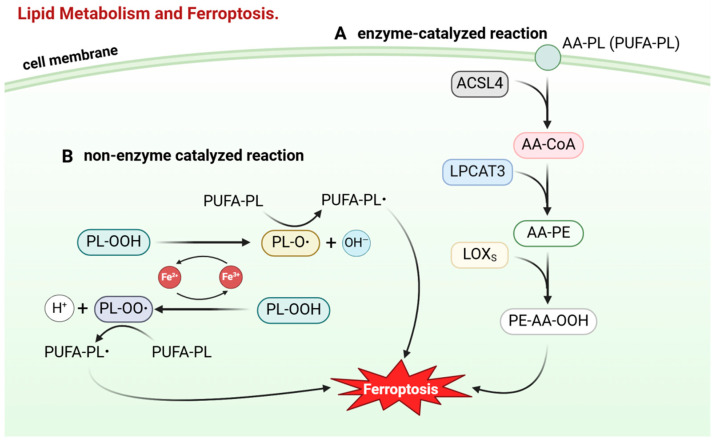
Lipid metabolism and ferroptosis: Lipid peroxides directly induce the occurrence of ferroptosis. The formation of lipid peroxides proceeds via two pathways: enzymatic and non-enzymatic reactions. (**A**) Enzymatic reaction: AA-PL in the cell membrane is catalyzed by acyl-CoA synthetase long-chain family member 4 (ACSL4) to form AA-CoA, which is then sequentially catalyzed by lysophosphatidylcholine acyltransferase 3 (LPCAT3) and lipoxygenases (LOXs) to generate lipid peroxides. (**B**) Non-enzymatic reaction: Iron ions undergo the Fenton reaction to produce lipid peroxides. Created in BioRender.com (accessed on 9 September 2024).

The formation of lipid peroxides occurs primarily through two pathways: enzymatic and non-enzymatic reactions. Initially, in the enzymatic pathway, ACSL4 catalyzes the conversion of arachidonic acid to AA-CoA. Subsequently, under the catalysis of lysophosphatidylcholine acyltransferase 3 (LPCAT3), AA-CoA is esterified to form phosphatidylethanolamine (PE) [87,88,89,90]. Then, with the catalysis of ALOX15, the hydroperoxy form (PE-AA-OOH) is generated. If there is an insufficient amount of glutathione peroxidase 4 (GPX4) and glutathione (GSH) involved in the reduction reaction, PE-AA-OOH acts as the initiating factor for ferroptosis [91]. Research indicates that the inhibition of ACSL4 can suppress the occurrence of ferroptosis. Overexpression of ACSL4 in cancer cells resistant to ferroptosis, such as LNCap and K562, significantly increases their sensitivity to this process [92,93]. Compared to normal tissues, ACSL4 is downregulated in breast cancer, bladder cancer, and lung cancer, suggesting that these cancer cells regulate lipid metabolism to inhibit ferroptosis. This finding highlights the critical role of ACSL4 in cancer cells and the potential of targeting this enzyme for therapeutic intervention [94] (Figure 4A).

The non-enzymatic reaction primarily triggers ferroptosis through the Fenton reaction, initiated by iron and ferrous ions. Specifically, both free ferrous and ferric ions can react with hydrogen peroxide to form free radicals. Phospholipid hydroperoxides can also participate in the Fenton reaction as substrates, forming lipid radicals and further reacting with polyunsaturated fatty acids to propagate the oxidation chain [84,95]. Simultaneously, ferrous ions enhance the enzymatic activity of ALOX15, promoting the formation of lipid peroxides. Intracellular iron primarily exists in the form of complexes such as heme and ferritin, which are relatively stable. A small amount of iron exists in a free form, highly reactive, and is known as the labile iron pool. Therefore, an increase in the labile iron pool, caused by factors such as enhanced ferritin degradation and increased iron uptake by transferrin, makes cells more susceptible to ferroptosis (Figure 4B).

### 4.2. Monounsaturated Fatty Acids and Ferroptosis

Not all lipid metabolism promotes ferroptosis in cells. Monounsaturated fatty acids refer to fatty acids containing one double bond. Monounsaturated fatty acids are believed to inhibit the occurrence of ferroptosis. Magtanong L et al. found that exogenous MUFA can inhibit the accumulation of cellular lipid ROS and reduce the peroxidation of PUFA, thereby inhibiting the occurrence of ferroptosis [96].

Studies have shown that when using different exogenous fatty acids as raw materials for tumor cell culture, polyunsaturated fatty acids (PUFAs) with strong peroxidative reactivity, including linoleic acid and arachidonic acid, promote ferroptosis in tumor cells. Conversely, monounsaturated fatty acids (MUFAs) with low peroxidative reactivity inhibit ferroptosis in these cells [90,97]. Furthermore, conjugated polyunsaturated fatty acids demonstrate a stronger ability to induce ferroptosis in tumors compared to non-conjugated polyunsaturated fatty acids [98]. Other studies indicate that certain lipids prone to peroxidation, such as 7-dehydrocholesterol and vitamin A, can also inhibit the occurrence of ferroptosis [99,100]. The conversion of polyunsaturated phospholipids (PUFA-PLs) and monounsaturated phospholipids (MUFA-PLs) in cells is determined by the content of polyunsaturated and monounsaturated fatty acids, as well as the phospholipid remodeling process (Lands’ cycle). Therefore, the cellular content of these fatty acids and the phospholipid remodeling process may also influence the sensitivity of cells to ferroptosis. Stearoyl-CoA desaturase (SCD) catalyzes the conversion of polyunsaturated fatty acids to monounsaturated fatty acids on the endoplasmic reticulum membrane. Studies have shown that the expression level of SCD in tumor cells is significantly increased compared to normal tissues, thereby reducing the occurrence of ferroptosis [101].

## 5. Metabolic Reprogramming and Ferroptosis in the Tumor Microenvironment

The tumor microenvironment (TME) refers to the environment surrounding a tumor, including the surrounding blood vessels, immune cells, fibroblasts, and extracellular matrix. The TME is closely related to the tumor, and they interact with each other. Tumor cells promote their own growth by influencing the microenvironment, while the body stimulates the elimination of tumors by altering the microenvironment [102]. In addition to metabolic reprogramming in tumor cells, the TME also undergoes metabolic reprogramming during tumor progression. On one hand, the presence of tumor cells and their metabolic alterations actively affect the oxygen and nutrient levels in the surrounding environment, thereby influencing the metabolic patterns of the microenvironment. On the other hand, metabolic reprogramming in cells within the TME represents an adaptive change that contributes to tumor immune evasion and promotes tumor progression. Fibroblasts and immune cells play crucial roles in the TME. It is generally believed that immune cells that promote tumor development are more reliant on lipid metabolism, while those that inhibit tumor development are more dependent on glucose metabolism [103]. Metabolic reprogramming of cells in the TME also affects ferroptosis, thereby influencing tumor initiation and progression. Therefore, understanding metabolic reprogramming in the TME is essential for gaining deeper insights into tumor metabolic reprogramming. Next, we will discuss the changes in metabolic reprogramming and ferroptosis in fibroblasts and immune cells within the TME, providing new insights for targeted metabolic anti-tumor therapies.

### 5.1. Fibroblast Metabolic Reprogramming and Ferroptosis

Cancer-associated fibroblasts (CAFs), a type of stromal cell in the tumor microenvironment, release bioactive substances that affect tumor cell metabolism and promote tumor cell proliferation [104]. To adapt to the tumor microenvironment, CAFs undergo metabolic reprogramming, including reprogramming of glucose, amino acid, and lipid metabolism. This metabolic reprogramming impacts the cellular oxidative stress status and ferroptosis.

In glucose metabolic reprogramming, CAFs exhibit enhanced glycolysis. Studies have shown that, compared to normal fibroblasts, breast cancer CAFs have highly methylated lactate dehydrogenase and pyruvate dehydrogenase genes, leading to increased expression of PKM2 and LDHA and promoting glycolysis [105]. Decreased expression of caveolin-1 (Cav-1) in CAFs activates TGF-β signaling and increases their glycolytic level [106]. Additionally, research indicates that ATM phosphorylates GLUT1 in CAFs, promoting glucose uptake and increasing glycolytic activity [107]. By enhancing glycolytic metabolism, CAFs produce and release metabolites such as lactate, which are taken up by tumor cells to promote their metabolism and proliferation. Studies have demonstrated that in prostate cancer, CAFs overexpress GLUT1 and MCT4, increasing glycolysis and lactate export rates, while prostate cancer cells show decreased GLUT1 expression, reduced glucose uptake, and increased lactate uptake [108]. This suggests that CAFs can promote tumor cell growth by releasing lactate. Furthermore, increased glycolysis in CAFs reduces ROS production, thereby decreasing oxidative stress and ferroptosis in both tumor and microenvironmental cells. Research has shown that CAFs can increase glutathione levels in tumor cells, enhancing prostate cancer resistance to cisplatin [109]. CAFs also secrete cytokines that affect tumor cell proliferation and metabolism. Studies indicate that CAFs can secrete IL-6 and IL-8, inducing phosphorylation of PGM1 in tumor cells and promoting glycolysis and the pentose phosphate pathway (PPP) [110]. This contributes to the inhibition of ferroptosis in tumor cells.

In amino acid metabolic reprogramming, CAFs synthesize and release amino acids required for tumor cell growth, promoting tumor progression. Research has shown that in ovarian cancer, CAFs overexpress glutamine synthetase (GS), producing more glutamine (Gln) that is taken up by cancer cells to support tumor growth [111]. As a substrate for glutathione synthesis, Gln produced by CAFs and taken up by tumor cells also increases glutathione synthesis in tumor cells, maintaining redox homeostasis and inhibiting ferroptosis.

In lipid metabolic reprogramming, CAFs exhibit increased lipid uptake and enhanced lipid metabolism. Studies have demonstrated that, compared to normal fibroblasts, CAFs overexpress fatty acid synthase (FASN), increasing the uptake and metabolism of fatty acids and phospholipids, and releasing lipid metabolites that promote the growth of colorectal cancer cells [112]. Additionally, research indicates that CAFs in lung cancer, through HIF-1α, increase the expression of stearoyl-CoA desaturase 1 (SCD1), thereby increasing the number of lipid droplets in cells and promoting lung cancer growth [113]. CAFs can also affect tumor cell lipid metabolism and ferroptosis through exosomes. Studies have shown that cisplatin and paclitaxel activate the USP7/hnRNPA1 axis to promote CAF secretion of miR-522, which inhibits the activity of ALOX15 in tumor cells, reduces ROS production, and inhibits ferroptosis in tumor cells [114].

### 5.2. Metabolic Reprogramming and Ferroptosis in Immune Cells

In the tumor microenvironment, immune cells play a crucial role. Antitumor immune cells include CD8+ T cells, NK cells, and M1 macrophages, while immune cells that promote tumor growth include Treg cells and M2 macrophages. The tumor microenvironment affects the metabolism and function of immune cells, which in turn influence the tumor microenvironment and tumor progression. Antitumor immune cells rely on glycolysis for their function, whereas immunoregulatory cells depend on lipid metabolism to exert tumor immunosuppressive effects [115]. We will specifically explore the relationship between metabolic changes in different immune cells and tumor development, and relate it to cellular ferroptosis, providing new insights for immunotherapy.

#### 5.2.1. CD8+ T Cells

CD8+ T cells primarily function to recognize and kill tumor cells, making them an important component of antitumor immune cells. However, as tumors progress, changes in the tumor microenvironment, such as hypoxia, nutrient depletion, and accumulation of tumor metabolites, can lead to a decrease in the antitumor activity of CD8+ T cells, converting them into a state of T cell exhaustion [116].

Alterations in glucose metabolism of CD8+ T cells affect their antitumor activity. CD8+ T cells require large amounts of glucose for glycolysis to meet the high energy demands of their antitumor activity. Due to glucose deficiency in the tumor microenvironment and competition for glucose by tumor cells, the glycolytic capacity of CD8+ T cells decreases, resulting in reduced cytokine production and antitumor ability [117]. Studies have shown that tumor cells produce and release lactate into the tumor microenvironment, inhibiting glycolysis and lactate production in CD8+ T cells, thereby suppressing their energy metabolism and antitumor effects [118]. Normally, moderate levels of ROS promote CD8+ T cell activation and cytokine production, but excessive ROS can inhibit CD8+ T cell antitumor activity or cause their death. Research indicates that a deficiency in electron transport chain complex III in CD8+ T cells leads to increased ROS production, causing CD8+ T cell death [119]. The tumor microenvironment, to some extent, promotes CD8+ T cell ferroptosis by increasing ROS, leading to immunosuppression and tumor progression. Studies have shown that defects in the *cdpdc5* promote ATF4 expression through mTORC1 signaling, causing CD8+ T cells to produce more ROS and undergo ferroptosis [120]. Additionally, enhanced PPP can enhance CD8+ T cell antitumor activity. Research indicates that CD8+ T cells in the tumor microenvironment can increase PPP metabolism by overexpressing PCK1, inhibiting tumor growth [121]. PPP metabolism is closely related to ferroptosis, and CD8+ T cells may inhibit ferroptosis through PPP to maintain their antitumor activity.

Amino acid metabolism plays a crucial role in regulating the antitumor function of CD8+ T cells. Limiting glutamine metabolism promotes the generation of memory T cells and increases antitumor activity. Studies have shown that CD8+ T cells cultured under glutamine-restricted conditions express more pro-survival factors and memory T cell differentiation transcription factors such as Tcf7, Lef1, and Bcl6, thereby promoting CD8+ T cell proliferation and antitumor activity [122]. The synthesis and function of GSH in CD8+ T cells are related to their survival, proliferation, and antitumor function. Research indicates that the A2AR signaling cascade promotes GSH depletion, leading to GSSG accumulation and lipid peroxidation, thereby inducing CD8+ T cell ferroptosis. Inhibiting A2AR can maintain CD8+ T cell activity and exert antitumor effects [123].

CD8+ T cells in the immune microenvironment undergo significant changes in lipid metabolism that affect their function. As tumors progress, the immune microenvironment becomes nutrient-poor, and to maintain cell survival, CD8+ T cells undergo metabolic adaptation, promoting lipid metabolism as an alternative energy source to glucose metabolism. However, excessive lipid metabolism can lead to ROS production and oxidative stress. Studies have shown that cholesterol accumulates in the tumor microenvironment and induces CD8+ T cell immune checkpoint expression, inhibiting their antitumor activity [124]. Excessive lipid metabolism can lead to CD8+ T cell exhaustion by increasing ferroptosis. Research indicates that the TME can cause lipid accumulation, particularly the accumulation of ox-LDL, leading to increased intracellular lipid peroxides and promoting CD8+ T cell ferroptosis, resulting in the inactivation of antitumor effects [125]. Inhibiting ferroptosis can enhance antitumor immunity. Studies have shown that degrading lipid peroxides through GPX4 can inhibit CD8+ T cell ferroptosis, thereby increasing the sensitivity to immunotherapy [126].

In summary, CD8+ T cells in the tumor microenvironment can affect the occurrence of ferroptosis by altering glucose metabolism, amino acid metabolism, and lipid metabolism, inhibiting their antitumor activity and promoting tumor initiation and progression.

#### 5.2.2. Treg Cells

In the tumor microenvironment, Treg cells exert immunosuppressive effects by secreting immunosuppressive cytokines such as IL-4, IL-10, and TGF-β, and expressing inhibitory cell surface receptors such as PD-1 and CTLA-4.

Regarding glucose metabolism, Treg cells preferentially undergo tricarboxylic acid cycle and oxidative phosphorylation metabolism under normal conditions. Due to nutrient deficiency in the tumor microenvironment, Treg cells reduce glycolysis. Studies have shown that the transcription factor Foxp3 can inhibit *myc* expression and suppress glycolysis in Treg cells [127]. Treg cells can be activated through TLR8, inhibiting their glucose uptake and glycolysis, leading to a reversal of their function [128]. However, some studies have found that some Treg cells still rely on glycolysis for metabolism and upregulate glucose transporters. Research indicates that in ovarian cancer, Treg cells upregulate the expression of glucose-metabolism-related proteins Glut1, Glut3, and HIF-1α [129]. In summary, Treg cells exhibit diverse metabolic changes and good metabolic adaptability, allowing them to survive in the tumor microenvironment and exert immunosuppressive effects.

The amino acid metabolic reprogramming of Treg cells promotes their survival in the immune microenvironment. Tumor cells overexpress SLC7A11, promoting the export of glutamate from the tumor, leading to glutamate accumulation in the tumor microenvironment. Glutamate promotes Treg proliferation and immunosuppressive effects [130]. Studies have shown that in metastatic melanoma, Treg cells overexpress ARG2, affecting arginine metabolism and promoting their immunosuppressive function, possibly through mTORC signaling [131]. Treg cells increase GSH synthesis to limit serine metabolism, scavenge ROS, ensure their stability, and maintain their immunosuppressive effects [132]. GSH inhibits the occurrence of ferroptosis, and Treg cells may maintain their immunosuppressive effects and promote tumor development by increasing GSH synthesis to inhibit ferroptosis.

In the tumor microenvironment, Treg cells enhance lipid metabolism to promote their proliferation and exert anti-immune effects. Genes related to lipid metabolism are significantly overexpressed in Treg cells in the tumor microenvironment [133]. Studies have shown that Treg cells can promote FASN-mediated de novo fatty acid synthesis through SCAP and SREBP signaling, maintaining their anti-immune effects [133]. Treg cells upregulate CD36 expression, increase FFA uptake, activate PPAR-β signaling, promote mitochondrial lipid metabolism and energy supply, and maintain their cell activity [134]. The high fatty acid metabolism and mitochondrial activity of Treg cells can lead to excessive ROS production and secretion into the tumor microenvironment, disrupting the activity of other immune cells [135]. The mechanism may involve excessive ROS in the tumor microenvironment promoting ferroptosis in other immune cells, leading to tumor immunosuppression.

#### 5.2.3. Tumor-Associated Macrophages (TAMs)

There are two phenotypes of tumor-associated macrophages: M1, which has antitumor effects, and M2, which promotes tumor growth. M1 macrophages synthesize and secrete inflammatory mediators using fatty acid metabolism and obtain most of their ATP through glycolysis, while M2 macrophages obtain their main ATP through fatty acid metabolism [136]. As tumors progress, fibroblasts secrete lipids and lipid metabolites into the tumor microenvironment, promoting the conversion of TAMs from M1 to M2 phenotype [137]. Studies have shown that M2 macrophages increase lipid oxidation metabolism, promote mitochondrial oxidative phosphorylation and ROS production, and promote the expression of genes that regulate TAM function [138]. The polarization of macrophages to the M2 phenotype is induced by lipid metabolism. Studies have shown that inhibiting fatty acid oxidation metabolism or the PPARγ signaling pathway in TAMs inhibits the generation of M2 macrophages and tumor development [139]. Changes in cholesterol metabolism in the tumor microenvironment are related to tumor ferroptosis. Studies have shown that overexpressing TMEM147 in hepatocellular carcinoma upregulates GPX4 activity by promoting DHCR7 expression, leading to tumor cell ferroptosis resistance and increased lipid metabolism in macrophages, thereby promoting their M2 polarization and exerting immunosuppressive effects [140].

#### 5.2.4. Dendritic Cells (DCs)

Dendritic cells are important antigen-presenting cells that can activate CD8+ T cell-mediated antitumor immune responses through antigen presentation. In the tumor microenvironment, increased lipid metabolism in DCs reduces their antigen-presenting ability and promotes tumor immune escape. Studies have shown that fatty acid receptors are significantly upregulated in tumor DCs, leading to increased intracellular lipid content [141]. Other studies have shown that tumor DCs can accumulate lipid peroxides through XBP1, inducing endoplasmic reticulum stress and inhibiting their antigen-presenting ability, thereby promoting tumor immune escape [142]. Tumor cells can secrete lipid exosomes (GDEs) to affect DC metabolism and induce ferroptosis. Studies have shown that inhibiting GDE secretion can reduce lipid peroxidation levels in glioblastoma DCs, decreasing DC ferroptosis and maintaining antitumor effects, specifically through the NRF2/GPX4 pathway [143].

#### 5.2.5. Natural Killer (NK) Cells

NK cells can release cytotoxic substances such as perforin and granzyme or directly kill tumor cells through antibody-dependent cell-mediated cytotoxicity (ADCC), exerting antitumor effects. NK cells also promote antitumor immunity by secreting cytokines. The metabolic alterations of NK cells in the tumor microenvironment impair their antitumor functions. Studies have shown that elevated expression of lactate dehydrogenase A (LDHA) in melanoma increases lactate synthesis and release, inhibiting the antitumor activity of NK cells and leading to tumor immune escape [144]. In the tumor microenvironment, glycolysis and oxidative phosphorylation of NK cells are inhibited. Research indicates that increased cholesterol metabolites, such as 27-hydroxycholesterol (27-HC), in the immune microenvironment reduce the expression of SLC25A1 and ACLY in NK cells, thereby inhibiting their glycolysis and oxidative phosphorylation and resulting in decreased antitumor function of NK cells [145]. Additionally, a lipid-rich tumor microenvironment causes lipid accumulation in NK cells, further inhibiting their antitumor activity [146]. Different cell types in the tumor microenvironment interact with each other. One study demonstrated that cancer-associated fibroblasts in gastric cancer promote iron export into the tumor microenvironment, increasing iron metabolism instability in NK cells, leading to NK cell ferroptosis and facilitating tumor immune escape [147].

The tumor microenvironment is complex in terms of cell types and metabolic components. Different cell types undergo distinct metabolic alterations, primarily to adapt to changes in the surrounding environment during tumor development and to maintain tumor progression through mutual interactions. Changes in the tumor microenvironment play a crucial role in inducing drug resistance in tumor treatment. The various metabolic alterations in the tumor microenvironment, particularly those in immune cells leading to tumor immune escape through multiple mechanisms, represent a significant challenge in tumor treatment. Targeting metabolic reprogramming to promote antitumor immunity may be a novel approach to enhance tumor treatment.

## 6. Discussion

This review comprehensively explores the intimate connection between tumor metabolic reprogramming and ferroptosis, with a particular focus on the pivotal roles of glucose, protein, and lipid metabolism in this process. Through in-depth analysis, we unveil how tumor cells adapt their metabolic pathways to microenvironmental changes, subsequently influencing the onset and progression of ferroptosis.

Glucose metabolism, as the primary source of energy supply, undergoes reprogramming that not only alters the energy status of tumor cells but also indirectly regulates ferroptosis sensitivity by affecting the redox balance. Reprogramming of protein metabolism, especially the uptake and utilization of amino acids as well as protein synthesis and degradation, not only meets the demands of rapid tumor proliferation but also directly participates in the signaling and regulation of ferroptosis, offering a new perspective for understanding the development of tumor resistance. Furthermore, the close link between lipid metabolism and ferroptosis, particularly the balance between the generation and clearance of lipid peroxides, highlights the potential of intervening in lipid metabolism to regulate lipid peroxide levels and thereby control the occurrence of ferroptosis, representing an innovative therapeutic strategy for cancer. Additionally, factors such as the interaction between immune cells and stromal cells within the tumor microenvironment may regulate metabolic pathways to influence tumor cells’ response to ferroptosis, emphasizing the significance of the tumor microenvironment in ferroptosis regulation and providing a novel theoretical basis for developing therapeutic strategies targeting the tumor microenvironment.

Future research should further investigate the dynamic changes in metabolic reprogramming and ferroptosis during tumor initiation, progression, and metastasis, and explore how to leverage these changes to enhance the therapeutic sensitivity of tumor cells. Moreover, the development of new drugs or therapeutic approaches that can specifically intervene in tumor metabolism and the ferroptosis process will be a crucial direction for future anti-cancer research. Through the integrated application of interdisciplinary knowledge and technological means, we are confident that more breakthroughs will be made in the field of tumor metabolism and ferroptosis, ultimately providing more effective treatment options for cancer patients.

## Data Availability

The data that support the findings of this study are openly available in public databases, which are included within the article.
